# Supplementary material for: Coenrollment of critically ill patients in PROSPECT: A protocol and statistical analysis plan
Source: PLoS One. 2026 Mar 23;21(3):e0345656. doi: 10.1371/journal.pone.0345656 (PMC13008073; doi:10.1371/journal.pone.0345656)
Supplement: S2 File — (PDF) [file pone.0345656.s002.pdf]

Supplemental Online Content: Nonauthor Collaborators

*Indicates required information. Only first name, last name, and suffix will appear in PubMed.

Group Name(s):* The PROSPECT Investigators and the Canadian Critical Care Trials Group members

*First Name and Middle Initial(s)	*Last Name	*Suffix (eg, Jr, III)	Academic Degrees	Institution	Location (city, state/province, country)	Role or Contribution, eg, chair, principal investigator	Group (if more than 1 Group listed in the byline) and/or Subgroup (eg, Steering Committee)
Christine	Wallace		BSc(Pharm), RPh	St. Joseph's Healthcare	Hamilton, Ontario, Canada	Pharmacy	Pharmacy Consultant
Gita	Sobhi		BSc(Pharm)	Hamilton Health Science Center	Hamilton, Ontario, Canada	Pharmacy	Pharmacy Consultant
Jennie	Johnstone		MD, PhD	St. Joseph`s Health Center	Toronto, Ontario, Canada	Adjudicator	Pneumonia and other Infection Adjudication Committee
François	Lauzier		MD, MSc, FRCPC	CHU de Québec-Université Laval (Hôpital de l’Enfant-Jésus	Québec City, Quebec, Canada	Adjudicator	Pneumonia and other Infection Adjudication Committee
Deborah	Cook		MD	St. Joseph`s Healthcare	Hamilton, Ontario, Canada	Adjudicator	Pneumonia and other Infection Adjudication Committee
Erick	Duan		MD, MSc	Niagara Health	St. Catharines, Ontario, Canada	Adjudicator	Pneumonia and other Infection Adjudication Committee
Joanna	Dionne		MD, MSc	Jurvinski Hospital	Hamilton, Ontario, Canada	Adjudicator	Pneumonia and other Infection Adjudication Committee
Bram	Rochwerg		MD	Jurvinski Hospital	Hamilton, Ontario, Canada	Adjudicator	Pneumonia and other Infection Adjudication Committee
John	Centoanti		MD	Hamilton General Hospital	Hamilton, Ontario, Canada	Adjudicator	Pneumonia and other Infection Adjudication Committee
Simon	Oczkowski		MD	McMaster University	Hamilton, Ontario, Canada	Adjudicator	Pneumonia and other Infection Adjudication Committee
Joanna	Dionne		MD, MSc	Jurvinski Hospital	Hamilton, Ontario, Canada	Adjudicator	Clostridioides Difficile Infection Adjudication Committee
Erick	Duan		MD, MSc	Niagara Health	St. Catharines, Ontario, Canada	Adjudicator	Clostridioides Difficile Infection Adjudication Committee
Jennie	Johnstone		MD, PhD	St. Joseph`s Health Center	Toronto, Ontario, Canada	Adjudicator	Clostridioides Difficile Infection Adjudication Committee
Daphne	Lamarche		PhD	McMaster University	Hamilton, Ontario, Canada	Centralized Laboratory	Translational Science Committee
Michael	Surette		PhD	McMaster University	Hamilton, Ontario, Canada	Centralized Laboratory	Translational Science Committee
Dawn	Bowdish		PhD	McMaster University	Hamilton, Ontario, Canada	Centralized Laboratory	Translational Science Committee
Andreas	Laupacis		MD, MSc, BA	University of Toronto, St. Michael`s Hospital	Toronto, Ontario, Canada	Data Monitoring Committee	Data Monitoring Committee (Lead)
Robin	Roberts		PhD	McMaster University	Hamilton, Ontario, Canada	Data Monitoring Committee	Data Monitoring Committee
Christian	Brun-Buisson		MD	Université Paris Est Créteil	Paris, France	Data Monitoring Committee	Data Monitoring Committee
Steve	Reynolds		MD	Royal Columbia Hospital	New Westminster, British Columbia, Canada	Site Investigator	
Sue	Willems		BScN, CCCRP	Royal Columbia Hospital	New Westminster, British Columbia, Canada	Research Coordinator	
Tina	Sekhon		BSc(Pharm)	Royal Columbia Hospital	New Westminster, British Columbia, Canada	Pharmacy	
Peter	Dodek		MD, MHSc	St. Paul's Hospital	Vancouver, British Columbia, Canada	Site Investigator	
Najib	Ayas		MD	St. Paul's Hospital	Vancouver, British Columbia, Canada	Site Co-Investigator	
Maria	Agda		BSc	St. Paul's Hospital	Vancouver, British Columbia, Canada	Research Coordinator	
Victoria	Alcuaz		BScN	St. Paul's Hospital	Vancouver, British Columbia, Canada	Research Coordinator	
Betty-Jean	Ashley		RN	St. Paul's Hospital	Vancouver, British Columbia, Canada	Research Coordinator	
Kelsey	Brewer			St. Paul's Hospital	Vancouver, British Columbia, Canada	Research Coordinator	
Janice	Palmer		BScN	St. Paul's Hospital	Vancouver, British Columbia, Canada	Research Coordinator	
Glen	Brown		PharmD	St. Paul's Hospital	Vancouver, British Columbia, Canada	Pharmacy	
Mara	Pavan		BSc(Pharm)	St. Paul's Hospital	Vancouver, British Columbia, Canada	Pharmacy	
William	Henderson		MD	Vancouver General Hospital	Vancouver, British Columbia, Canada	Site Investigator	
Donald	Greisdale		MD, MPH	Vancouver General Hospital	Vancouver, British Columbia, Canada	Site Co-Investigator	
Mypinder	Sekhon		MD, PhD	Vancouver General Hospital	Vancouver, British Columbia, Canada	Site Co-Investigator	
Denise	Foster		RN	Vancouver General Hospital	Vancouver, British Columbia, Canada	Research Coordinator	
Suzie	Logie		CCRN, CCRP	Vancouver General Hospital	Vancouver, British Columbia, Canada	Research Coordinator	
Judy	Yip		BSc(Pharm)	Vancouver General Hospital	Vancouver, British Columbia, Canada	Pharmacy	
Gordon	Wood		MD, FRCPC	Vancouver Island Health Authority	Victoria, British Columbia, Canada	Site Investigator	
Daniel	Ovakim		MD, MSc, FRCPC	Vancouver Island Health Authority	Victoria, British Columbia, Canada	Site Co-Investigator	
Fiona	Auld		BScN, CCRP	Vancouver Island Health Authority	Victoria, British Columbia, Canada	Research Coordinator	
Gayle	Carney		BScN, CCRN	Vancouver Island Health Authority	Victoria, British Columbia, Canada	Research Coordinator	
Ralph	Fleming			Vancouver Island Health Authority	Victoria, British Columbia, Canada	Research Coordinator	
Jennifer	Good		BScN	Vancouver Island Health Authority	Victoria, British Columbia, Canada	Research Coordinator	
Mandeep	Manhas		MBBS, MSc, CCRP	Vancouver Island Health Authority	Victoria, British Columbia, Canada	Research Coordinator	
Karin	Boyd			Vancouver Island Health Authority	Victoria, British Columbia, Canada	Pharmacy	
Jane	Dheere		BSc(Pharm)	Vancouver Island Health Authority	Victoria, British Columbia, Canada	Pharmacy	
Tom	Stelfox		MD	Foothills Hospital, Univeristy of Calgary	Calgary, Alberta, Canada	Site Investigator	
Philippe	Couillard		MD	Foothills Hospital, Univeristy of Calgary	Calgary, Alberta, Canada	Site Co-Investigator	
Christopher	Doig		MD	Foothills Hospital, Univeristy of Calgary	Calgary, Alberta, Canada	Site Co-Investigator	
Ken	Parhar		MD	Foothills Hospital, Univeristy of Calgary	Calgary, Alberta, Canada	Site Co-Investigator	
Joshua	Booth		BA	Foothills Hospital, Univeristy of Calgary	Calgary, Alberta, Canada	Research Coordinator	
Cassidy	Codan		BSc	Foothills Hospital, Univeristy of Calgary	Calgary, Alberta, Canada	Research Coordinator	
Stacy	Ruddell		MSc	Foothills Hospital, Univeristy of Calgary	Calgary, Alberta, Canada	Research Coordinator	
Candice	Cameron		BSP	Foothills Hospital, Univeristy of Calgary	Calgary, Alberta, Canada	Pharmacy	
Rhonda	Edison		RPhT	Foothills Hospital, Univeristy of Calgary	Calgary, Alberta, Canada	Pharmacy	

*Indicates required information. Only first name, last name, and suffix will appear in PubMed.

***First Name and Middle Initial(s)**	***Last Name**	***Suffix (eg, Jr, III)**	Academic Degrees	Institution	Location (city, state/province, country)	Role or Contribution, eg, chair, principal investigator	Group (if more than 1 Group listed in the byline) and/or Subgroup (eg, Steering Committee)	
Anne	Martin		RPhT	Foothills Hospital, Univeristy of Calgary	Calgary, Alberta, Canada	Pharmacy		
Breanna	Mina		RPhT	Foothills Hospital, Univeristy of Calgary	Calgary, Alberta, Canada	Pharmacy		
Daniel	Niven		MD	Peter Lougheed Center, University of Calgary	Calgary, Alberta, Canada	Site Investigator		
Luc	Berthiaume		MD	Peter Lougheed Center, University of Calgary	Calgary, Alberta, Canada	Site Co-Investigator		
Jonathan	Gaudet		MD	Peter Lougheed Center, University of Calgary	Calgary, Alberta, Canada	Site Co-Investigator		
Joshua	Booth		BA	Peter Lougheed Center, University of Calgary	Calgary, Alberta, Canada	Research Coordinator		
Cassidy	Codan		BSc	Peter Lougheed Center, University of Calgary	Calgary, Alberta, Canada	Research Coordinator		
Stacy	Ruddell		MSc	Peter Lougheed Center, University of Calgary	Calgary, Alberta, Canada	Research Coordinator		
Candice	Cameron		BSP	Peter Lougheed Center, University of Calgary	Calgary, Alberta, Canada	Pharmacy		
Rhonda	Edison		RPhT	Peter Lougheed Center, University of Calgary	Calgary, Alberta, Canada	Pharmacy		
Anne	Martin		RPhT	Peter Lougheed Center, University of Calgary	Calgary, Alberta, Canada	Pharmacy		
Breanna	Mina		RPhT	Peter Lougheed Center, University of Calgary	Calgary, Alberta, Canada	Pharmacy		
Jim	Kutsiogiannis		MD, MHSc, FRCPC	Royal Alexandra Hospital	Edmonton, Alberta, Canada	Site Investigator		
Raiyan	Chowdhury		MD, MHSc, FRCPC	Royal Alexandra Hospital	Edmonton, Alberta, Canada	Site Co-Investigator		
Jon	Davidow		MD, FRCPC	Royal Alexandra Hospital	Edmonton, Alberta, Canada	Site Co-Investigator		
Curt	Johnston		MD, FRCPC	Royal Alexandra Hospital	Edmonton, Alberta, Canada	Site Co-Investigator		
Richard	Johnston		MD, MBA	Royal Alexandra Hospital	Edmonton, Alberta, Canada	Site Co-Investigator		
Kim	Macala		MD, FRCPC, PhD	Royal Alexandra Hospital	Edmonton, Alberta, Canada	Site Co-Investigator		
Sam	Marcushamer		MD, FRCPC	Royal Alexandra Hospital	Edmonton, Alberta, Canada	Site Co-Investigator		
Darren	Markland		MD, FRCPC	Royal Alexandra Hospital	Edmonton, Alberta, Canada	Site Co-Investigator		
Doug	Matheson		MD, FRCPC	Royal Alexandra Hospital	Edmonton, Alberta, Canada	Site Co-Investigator		
Damian	Payton-Gay		MD, FRCPC	Royal Alexandra Hospital	Edmonton, Alberta, Canada	Site Co-Investigator		
David	Zygun		MD, FRCPC, MSc, MH	Royal Alexandra Hospital	Edmonton, Alberta, Canada	Site Co-Investigator		
Tayne	Hewer		MSc	Royal Alexandra Hospital	Edmonton, Alberta, Canada	Research Coordinator		
Pat	Thompson		RN	Royal Alexandra Hospital	Edmonton, Alberta, Canada	Research Coordinator		
Maggie	Ge		RPhT	Royal Alexandra Hospital	Edmonton, Alberta, Canada	Pharmacy		
Janny	Hall		RPhT	Royal Alexandra Hospital	Edmonton, Alberta, Canada	Pharmacy		
Sharon	Matenchuk		BSc(Pharm), RPh	Royal Alexandra Hospital	Edmonton, Alberta, Canada	Pharmacy		
Wendy	Sligl		MD, MSc, FRCPC	University of Edmonton Hospital	Edmonton, Alberta, Canada	Site Investigator		
Sean	Bagshaw		MD, MSc, FRCPC	University of Edmonton Hospital	Edmonton, Alberta, Canada	Site Co-Investigator		
Nadia	Baig		BSc	University of Edmonton Hospital	Edmonton, Alberta, Canada	Research Coordinator		
Lorena	McCoshen		GN	University of Edmonton Hospital	Edmonton, Alberta, Canada	Research Coordinator		
Katrina	Alexandropoulos		RPhT	University of Edmonton Hospital	Edmonton, Alberta, Canada	Pharmacy		
Sherri	Bain		BSc(Pharm)	University of Edmonton Hospital	Edmonton, Alberta, Canada	Pharmacy		
Michelle	Brandt		BSc(Pharm)	University of Edmonton Hospital	Edmonton, Alberta, Canada	Pharmacy		
Cathy	Constable		RPhT	University of Edmonton Hospital	Edmonton, Alberta, Canada	Pharmacy		
Kari	Douglas		RPhT	University of Edmonton Hospital	Edmonton, Alberta, Canada	Pharmacy		
Shaleen	Maharaj		RPhT	University of Edmonton Hospital	Edmonton, Alberta, Canada	Pharmacy		
Sabrina	Travers		RPhT	University of Edmonton Hospital	Edmonton, Alberta, Canada	Pharmacy		
Ryan	Zarychanski		MD	Health Science Center	Winnipeg, Manitoba, Canada	Site Investigator		
Bojan	Paunovic		MD	Health Science Center	Winnipeg, Manitoba, Canada	Site Co-Investigator		
Justin	Lys		MD	Health Science Center	Winnipeg, Manitoba, Canada	Research Coordinator		
Nicole	Marten		RN	Health Science Center	Winnipeg, Manitoba, Canada	Research Coordinator		
Audrey	Bhairo			Health Science Center	Winnipeg, Manitoba, Canada	Pharmacy		
Halyna	Ferenes		RPEBC-PT	Health Science Center	Winnipeg, Manitoba, Canada	Pharmacy		
Beata	Kozak		BSc	Health Science Center	Winnipeg, Manitoba, Canada	Pharmacy		
Debra	Verrier		PA	Health Science Center	Winnipeg, Manitoba, Canada	Pharmacy		
Dawn-Lee	McLaughlin		PA	Health Science Center	Winnipeg, Manitoba, Canada	Pharmacy		
Maria	Valente		PA	Health Science Center	Winnipeg, Manitoba, Canada	Pharmacy		
Bojan	Paunovic		MD	St. Boniface Hospital	Winnipeg, Manitoba, Canada	Site Investigator		
Ryan	Zarychanski		MD	St. Boniface Hospital	Winnipeg, Manitoba, Canada	Site Co-Investigator		
Marcus	Blouw		MD	St. Boniface Hospital	Winnipeg, Manitoba, Canada	Site Co-Investigator		
Kendiss	Olafson		MD	St. Boniface Hospital	Winnipeg, Manitoba, Canada	Site Co-Investigator		
Heather	Smith		MD	St. Boniface Hospital	Winnipeg, Manitoba, Canada	Site Co-Investigator		
Oliver	Gutieror		MD	St. Boniface Hospital	Winnipeg, Manitoba, Canada	Research Coordinator		
Justin	Lys		MD	St. Boniface Hospital	Winnipeg, Manitoba, Canada	Research Coordinator		

*Indicates required information. Only first name, last name, and suffix will appear in PubMed.

*First Name and Middle Initial(s)	*Last Name	*Suffix (eg, Jr, III)	Academic Degrees	Institution	Location (city, state/province, country)	Role or Contribution, eg, chair, principal investigator	Group (if more than 1 Group listed in the byline) and/or Subgroup (eg, Steering Committee)	
Nicole	Marten		RN	St. Boniface Hospital	Winnipeg, Manitoba, Canada	Research Coordinator		
Sherri Lynn	Wingfield		BA	St. Boniface Hospital	Winnipeg, Manitoba, Canada	Research Coordinator		
Marnie	Boyle		BSc(Pharm)	St. Boniface Hospital	Winnipeg, Manitoba, Canada	Pharmacy		
Halyna	Ferenes		BSc(Pharm)	St. Boniface Hospital	Winnipeg, Manitoba, Canada	Pharmacy		
Debbie	Hrabi		RPEBC-PT	St. Boniface Hospital	Winnipeg, Manitoba, Canada	Pharmacy		
Beata	Kozak		BSc(Pharm)	St. Boniface Hospital	Winnipeg, Manitoba, Canada	Pharmacy		
Chantal	MacDonald		RPEBC-PT	St. Boniface Hospital	Winnipeg, Manitoba, Canada	Pharmacy		
Brenda	Reeve		MD, FRCPC	Brantford General Hospital	Brantford, Ontario Canada	Site Investigator		
Karen	Bento		BScN	Brantford General Hospital	Brantford, Ontario Canada	Research Coordinator		
Megan	Davis		BSc	Brantford General Hospital	Brantford, Ontario Canada	Research Coordinator		
William	Dechert		MSc	Brantford General Hospital	Brantford, Ontario Canada	Research Coordinator		
Barbara	Longo		BScN	Brantford General Hospital	Brantford, Ontario Canada	Research Coordinator		
Courtney	Mullen		HBSc	Brantford General Hospital	Brantford, Ontario Canada	Research Coordinator		
Elysia	Skrzpek		BScN	Brantford General Hospital	Brantford, Ontario Canada	Research Coordinator		
Laurenne	Wierenga		BScN	Brantford General Hospital	Brantford, Ontario Canada	Research Coordinator		
Wesam	Abuzaiter		BSc(Pharm)	Brantford General Hospital	Brantford, Ontario Canada	Pharmacy		
Lynda	Amorim		RPhT	Brantford General Hospital	Brantford, Ontario Canada	Pharmacy		
Rosemarie	Bauer		RPhT	Brantford General Hospital	Brantford, Ontario Canada	Pharmacy		
Rachel	Damota		RPhT	Brantford General Hospital	Brantford, Ontario Canada	Pharmacy		
Hoang	Thoa Ho		PharmD	Brantford General Hospital	Brantford, Ontario Canada	Pharmacy		
Nicole	Macdougall		RPhT	Brantford General Hospital	Brantford, Ontario Canada	Pharmacy		
Mary	Thornewell		BSC(Pharm), ACPR	Brantford General Hospital	Brantford, Ontario Canada	Pharmacy		
Lara	Tran		BSc(Pharm)	Brantford General Hospital	Brantford, Ontario Canada	Pharmacy		
Jennifer	Visocchi		BSC(Pharm), ACPR	Brantford General Hospital	Brantford, Ontario Canada	Pharmacy		
Paul	Hosek		MD, FRCPC	Grand River Hospital	Kitchener, Ontario, Canada	Site Investigator		
Bill	Plaxton		MD, FRCPC	Grand River Hospital	Kitchener, Ontario, Canada	Site Co-Investigator		
Catherine	Armstong		BScN	Grand River Hospital	Kitchener, Ontario, Canada	Research Coordinator		
William	Dechert		MSc	Grand River Hospital	Kitchener, Ontario, Canada	Research Coordinator		
Janelle	Ellis		BScN	Grand River Hospital	Kitchener, Ontario, Canada	Research Coordinator		
Kayla	Fisk		BScN	Grand River Hospital	Kitchener, Ontario, Canada	Research Coordinator		
Melissa	Gabnouri		RN	Grand River Hospital	Kitchener, Ontario, Canada	Research Coordinator		
Emilie	Gordon		BScN	Grand River Hospital	Kitchener, Ontario, Canada	Research Coordinator		
Rebecca	Haegens			Grand River Hospital	Kitchener, Ontario, Canada	Research Coordinator		
Lisa	Halford		BASc	Grand River Hospital	Kitchener, Ontario, Canada	Research Coordinator		
Brooklyn	Hillis		BScN	Grand River Hospital	Kitchener, Ontario, Canada	Research Coordinator		
Rebecca	Jesso		MScN, MN, NP	Grand River Hospital	Kitchener, Ontario, Canada	Research Coordinator		
Jennifer	McLaren		BScN, RN, CNCC(C)	Grand River Hospital	Kitchener, Ontario, Canada	Research Coordinator		
Elliot	McMillan		PhD	Grand River Hospital	Kitchener, Ontario, Canada	Research Coordinator		
Mariska	Pelkmans		BScN	Grand River Hospital	Kitchener, Ontario, Canada	Research Coordinator		
Matthew	Rekman		BScN	Grand River Hospital	Kitchener, Ontario, Canada	Research Coordinator		
Sylvia	Sinkovitis		BScN	Grand River Hospital	Kitchener, Ontario, Canada	Research Coordinator		
Monica	Truong		BScN	Grand River Hospital	Kitchener, Ontario, Canada	Research Coordinator		
Michelle	White		BSN, MN	Grand River Hospital	Kitchener, Ontario, Canada	Research Coordinator		
Noah	Bates		BSc-RPh	Grand River Hospital	Kitchener, Ontario, Canada	Pharmacy		
Susan	Bryden-Cromwell		BSc-RPh	Grand River Hospital	Kitchener, Ontario, Canada	Pharmacy		
Colleen	Cameron		PharmD	Grand River Hospital	Kitchener, Ontario, Canada	Pharmacy		
Aminah	Deen		DipPharmT	Grand River Hospital	Kitchener, Ontario, Canada	Pharmacy		
Sheri	DiGiovanni		BSc-RPh	Grand River Hospital	Kitchener, Ontario, Canada	Pharmacy		
Anders	Foss		BSc-RPh	Grand River Hospital	Kitchener, Ontario, Canada	Pharmacy		
Esther	Lee		BSc-RPh	Grand River Hospital	Kitchener, Ontario, Canada	Pharmacy		
Heidi	MacGregor		DipPharmT	Grand River Hospital	Kitchener, Ontario, Canada	Pharmacy		
Esther	Galbraith		BSC(Pharm)	Grand River Hospital	Kitchener, Ontario, Canada	Pharmacy		
Robyn	McArthur		PharmD	Grand River Hospital	Kitchener, Ontario, Canada	Pharmacy		
Julie	McGregor		DipPharmT	Grand River Hospital	Kitchener, Ontario, Canada	Pharmacy		
Keith	Miller		BSc-RPh	Grand River Hospital	Kitchener, Ontario, Canada	Pharmacy		
Sharon	Morris		DipPharmT	Grand River Hospital	Kitchener, Ontario, Canada	Pharmacy		

*First Name and Middle Initial(s)	*Last Name	*Suffix (eg, Jr, III)	Academic Degrees	Institution	Location (city, state/province, country)	Role or Contribution, eg, chair, principal investigator	Group (if more than 1 Group listed in the byline) and/or Subgroup (eg, Steering Committee)	
Shelley	Parker		BSc(Pharm), RPh	Grand River Hospital	Kitchener, Ontario, Canada	Pharmacy		
Candice	Smith		BSc-RPh	Grand River Hospital	Kitchener, Ontario, Canada	Pharmacy		
Joanna	Stoglow		DipBA	Grand River Hospital	Kitchener, Ontario, Canada	Pharmacy		
Jennifer	Tung		PharmD	Grand River Hospital	Kitchener, Ontario, Canada	Pharmacy		
Melissa	Vos		BSc(Pharm)	Grand River Hospital	Kitchener, Ontario, Canada	Pharmacy		
Maureen	Meade		MD, MSc	Hamilton General Hospital	Hamilton, Ontario, Canada	Site Investigator		
Emilie	Belley-Cote		MD, PhD	Hamilton General Hospital	Hamilton, Ontario, Canada	Site Co-Investigator		
Lori	Hand		RRT	Hamilton General Hospital	Hamilton, Ontario, Canada	Research Coordinator		
Lisa	Klotz		BSc	Hamilton General Hospital	Hamilton, Ontario, Canada	Research Coordinator		
Alexandra	Sabev		BA	Hamilton General Hospital	Hamilton, Ontario, Canada	Research Coordinator		
Nevena	Savija		MSc	Hamilton General Hospital	Hamilton, Ontario, Canada	Research Coordinator		
Paige	Guyatt		BA, BSc	Hamilton General Hospital	Hamilton, Ontario, Canada	Research Coordinator		
Deanna	Cosentino		RPhT	Hamilton General Hospital	Hamilton, Ontario, Canada	Pharmacy		
Diane	Lourenco		RPhT	Hamilton General Hospital	Hamilton, Ontario, Canada	Pharmacy		
Julie	Misina		RPhT	Hamilton General Hospital	Hamilton, Ontario, Canada	Pharmacy		
Gita	Sobhi		BSc(Pharm)	Hamilton General Hospital	Hamilton, Ontario, Canada	Pharmacy		
Paul	Lysecki		MD	Joseph Brant Hospital	Burlington, Ontario, Canada	Site Investigator		
Joseph	Berlingieri		MD	Joseph Brant Hospital	Burlington, Ontario, Canada	Site Co-Investigator		
Sameer	Shaikh		MD	Joseph Brant Hospital	Burlington, Ontario, Canada	Site Co-Investigator		
Steven	Skitch		MD, PhD	Joseph Brant Hospital	Burlington, Ontario, Canada	Site Co-Investigator		
Tracy	Campbell		MSc, RD	Joseph Brant Hospital	Burlington, Ontario, Canada	Research Coordinator		
Hala	Basheer		PharmD	Joseph Brant Hospital	Burlington, Ontario, Canada	Pharmacy		
Kathy	Bruder		RPhT	Joseph Brant Hospital	Burlington, Ontario, Canada	Pharmacy		
Jane	Cheng		BSc(Pharm)	Joseph Brant Hospital	Burlington, Ontario, Canada	Pharmacy		
Kaiser	Qureshi		BSc(Pharm). ACPR,	Joseph Brant Hospital	Burlington, Ontario, Canada	Pharmacy		
Celeste	Infantino		RPhT	Joseph Brant Hospital	Burlington, Ontario, Canada	Pharmacy		
Timothy	Karachi		MD	Jurvinski Hospital	Hamilton, Ontario, Canada	Site Investigator		
Bram	Rochwerg		MD	Jurvinski Hospital	Hamilton, Ontario, Canada	Site Investigator		
Mashari	Alghuroba		BSc	Jurvinski Hospital	Hamilton, Ontario, Canada	Research Coordinator		
Alia	Khaled		BSc	Jurvinski Hospital	Hamilton, Ontario, Canada	Research Coordinator		
Tina	Millen		RRT	Jurvinski Hospital	Hamilton, Ontario, Canada	Research Coordinator		
Ryan	Vaisler		MD	Jurvinski Hospital	Hamilton, Ontario, Canada	Research Coordinator		
Maya	Biljan		RPhT	Jurvinski Hospital	Hamilton, Ontario, Canada	Pharmacy		
Deane	Cosentino		RPhT	Jurvinski Hospital	Hamilton, Ontario, Canada	Pharmacy		
Brittany	Marriott		RPhT	Jurvinski Hospital	Hamilton, Ontario, Canada	Pharmacy		
Gita	Sobhi		MSc	Jurvinski Hospital	Hamilton, Ontario, Canada	Pharmacy		
John	Muscedere		MD	Kingston General Hospital	Kingston, Ontario, Canada	Site Investigator		
Gordon	Boyd		MD, PhD	Kingston General Hospital	Kingston, Ontario, Canada	Site Co-Investigator		
Christine	D`Arsigny		MD	Kingston General Hospital	Kingston, Ontario, Canada	Site Co-Investigator		
John	Drover		MD	Kingston General Hospital	Kingston, Ontario, Canada	Site Co-Investigator		
Jason	Erb		MD	Kingston General Hospital	Kingston, Ontario, Canada	Site Co-Investigator		
David	Maslove		MD	Kingston General Hospital	Kingston, Ontario, Canada	Site Co-Investigator		
Chris	Parker		MD	Kingston General Hospital	Kingston, Ontario, Canada	Site Co-Investigator		
Stephanie	Sibley		MD	Kingston General Hospital	Kingston, Ontario, Canada	Site Co-Investigator		
Tracy	Boyd		MSc	Kingston General Hospital	Kingston, Ontario, Canada	Research Coordinator		
Ilinca	Georgescu		MSc	Kingston General Hospital	Kingston, Ontario, Canada	Research Coordinator		
Miranda	Hunt		BA	Kingston General Hospital	Kingston, Ontario, Canada	Research Coordinator		
Danielle	Muscedere			Kingston General Hospital	Kingston, Ontario, Canada	Research Coordinator		
Cathy	Baker		RPhT	Kingston General Hospital	Kingston, Ontario, Canada	Pharmacy		
Jennifer	Engel		RPhT	Kingston General Hospital	Kingston, Ontario, Canada	Pharmacy		
Jennifer	Fleming		RPhT	Kingston General Hospital	Kingston, Ontario, Canada	Pharmacy		
Lisa	Roderick		RPhT	Kingston General Hospital	Kingston, Ontario, Canada	Pharmacy		
Shelley	Silk		RPhT	Kingston General Hospital	Kingston, Ontario, Canada	Pharmacy		
Marcy	Spencer		RPhT	Kingston General Hospital	Kingston, Ontario, Canada	Pharmacy		
Michelle	Tryon		BSc(Pharm)	Kingston General Hospital	Kingston, Ontario, Canada	Pharmacy		
Dave	Nagpal		MD, MSc	London Health Science Centre - University Hospital	London, Ontario, Canada	Site Investigator		
Ian	Ball		MD, MSc	London Health Science Centre - University Hospital	London, Ontario, Canada	Site Co-Investigator		

*Indicates required information. Only first name, last name, and suffix will appear in PubMed.

*First Name and Middle Initial(s)	*Last Name	*Suffix (eg, Jr, III)	Academic Degrees	Institution	Location (city, state/province, country)	Role or Contribution, eg, chair, principal investigator	Group (if more than 1 Group listed in the byline) and/or Subgroup (eg, Steering Committee)	
Michael	Sharpe		MD	London Health Science Centre - University Hospital	London, Ontario, Canada	Site Co-Investigator		
Karen J	Bosma		MD	London Health Science Centre - University Hospital	London, Ontario, Canada	Site Co-Investigator		
Tracey	Bentall			London Health Science Centre - University Hospital	London, Ontario, Canada	Research Coordinator		
Jessica	Sturt-Smith			London Health Science Centre - University Hospital	London, Ontario, Canada	Research Coordinator		
Michelle	Alexander		RPhT	London Health Science Centre - University Hospital	London, Ontario, Canada	Pharmacy		
Tammy	Ellis			London Health Science Centre - University Hospital	London, Ontario, Canada	Pharmacy		
Mindy	Muylaert		RPhT	London Health Science Centre - University Hospital	London, Ontario, Canada	Pharmacy		
Cindy	Paczkowski		RPhT	London Health Science Centre - University Hospital	London, Ontario, Canada	Pharmacy		
Ian	Ball		MD, MSc	London Health Science Centre - Victoria Hospital	London, Ontario, Canada	Site Investigator		
Eileen	Campbell			London Health Science Centre - Victoria Hospital	London, Ontario, Canada	Research Coordinator		
Susie	Imervoski		RRT	London Health Science Centre - Victoria Hospital	London, Ontario, Canada	Research Coordinator		
Athena	Ovsenek		BA	London Health Science Centre - Victoria Hospital	London, Ontario, Canada	Research Coordinator		
Rebecca	Rondinelli			London Health Science Centre - Victoria Hospital	London, Ontario, Canada	Research Coordinator		
Teresa	Longfield		RPhT	London Health Science Centre - Victoria Hospital	London, Ontario, Canada	Pharmacy		
Amy	Moyer		RPhT	London Health Science Centre - Victoria Hospital	London, Ontario, Canada	Pharmacy		
Faith	Norris		RPhT	London Health Science Centre - Victoria Hospital	London, Ontario, Canada	Pharmacy		
Janice	Sumpton		RPh	London Health Science Centre - Victoria Hospital	London, Ontario, Canada	Pharmacy		
Karina	Teterycz		RPhT	London Health Science Centre - Victoria Hospital	London, Ontario, Canada	Pharmacy		
Sangeeta	Mehta		MD	Mount Sinai Hospital	Toronto, Ontario, Canada	Site Investigator		
Stephen	Lapinsky		MD	Mount Sinai Hospital	Toronto, Ontario, Canada	Site Co-Investigator		
Laveena	Munshi		MD, MSc	Mount Sinai Hospital	Toronto, Ontario, Canada	Site Co-Investigator		
Maedean	Brown		MMHSc	Mount Sinai Hospital	Toronto, Ontario, Canada	Research Coordinator		
Brittany	Giacomino		RRT	Mount Sinai Hospital	Toronto, Ontario, Canada	Research Coordinator		
Marnie	Jakab		MD	Mount Sinai Hospital	Toronto, Ontario, Canada	Research Coordinator		
Alan	Kraguiljac		MSc	Mount Sinai Hospital	Toronto, Ontario, Canada	Research Coordinator		
Sumesh	Shah		CCRP	Mount Sinai Hospital	Toronto, Ontario, Canada	Research Coordinator		
Erik	Tamberg		BSc	Mount Sinai Hospital	Toronto, Ontario, Canada	Research Coordinator		
Doret	Cheng		PharmD	Mount Sinai Hospital	Toronto, Ontario, Canada	Pharmacy		
Gagan	Grewal		MSc	Mount Sinai Hospital	Toronto, Ontario, Canada	Pharmacy		
Andrew	Han		BSc(Pharm)	Mount Sinai Hospital	Toronto, Ontario, Canada	Pharmacy		
Holly	Leung		MSc	Mount Sinai Hospital	Toronto, Ontario, Canada	Pharmacy		
Ioanna	Mantas		BSc(Pharm)	Mount Sinai Hospital	Toronto, Ontario, Canada	Pharmacy		
Hilary	Rodrigues		BSc(Pharm	Mount Sinai Hospital	Toronto, Ontario, Canada	Pharmacy		
Andew	Wyllie		PharmD	Mount Sinai Hospital	Toronto, Ontario, Canada	Pharmacy		
Jennifer LY	Tsang		MD, PhD	Niagara Health	St. Catharines, Ontario, Canada	Site Investigator		
Erick	Duan		MD, MSc	Niagara Health	St. Catharines, Ontario, Canada	Site Co-Investigator		
Mercedes	Carmargo		MD, MSc	Niagara Health	St. Catharines, Ontario, Canada	Research Coordinator		
Beverly	Richter		RN	Niagara Health	St. Catharines, Ontario, Canada	Research Coordinator		
Rita	Caporuscio		BSc(Pharm)	Niagara Health	St. Catharines, Ontario, Canada	Pharmacy		
Rachel	Kressner Falvo		RPhT	Niagara Health	St. Catharines, Ontario, Canada	Pharmacy		
Dimitra	Fleming		BSc(Pharm)	Niagara Health	St. Catharines, Ontario, Canada	Pharmacy		
Carmelina	Maxwell		RPhT	Niagara Health	St. Catharines, Ontario, Canada	Pharmacy		
Karmen	Plantic		RPhT	Niagara Health	St. Catharines, Ontario, Canada	Pharmacy		
Lauralyn	McIntyre		MD	Ottawa Civic Hospital	Ottawa, Ontario, Canada	Site Investigator		
Giuseppe	Pagliarello		MD	Ottawa Civic Hospital	Ottawa, Ontario, Canada	Site Co-Investigator		
Gianni	D`Egidio		MD	Ottawa Civic Hospital	Ottawa, Ontario, Canada	Site Co-Investigator		
Shane	English		MD	Ottawa Civic Hospital	Ottawa, Ontario, Canada	Site Co-Investigator		
Mike	Hartwick		MD	Ottawa Civic Hospital	Ottawa, Ontario, Canada	Site Co-Investigator		
Jonathan	Hooper		MD	Ottawa Civic Hospital	Ottawa, Ontario, Canada	Site Co-Investigator		
Dal	Kubelik		MD	Ottawa Civic Hospital	Ottawa, Ontario, Canada	Site Co-Investigator		
Kwadwo	Kyeremanteng		MD	Ottawa Civic Hospital	Ottawa, Ontario, Canada	Site Co-Investigator		
Hilary	Meggison		MD	Ottawa Civic Hospital	Ottawa, Ontario, Canada	Site Co-Investigator		
David	Neilipovitz		MD	Ottawa Civic Hospital	Ottawa, Ontario, Canada	Site Co-Investigator		
Rakesh	Patel		MD	Ottawa Civic Hospital	Ottawa, Ontario, Canada	Site Co-Investigator		
Joseph	Po		MD	Ottawa Civic Hospital	Ottawa, Ontario, Canada	Site Co-Investigator		
Erin	Rosenberg		MD	Ottawa Civic Hospital	Ottawa, Ontario, Canada	Site Co-Investigator		

*Indicates required information. Only first name, last name, and suffix will appear in PubMed.

*First Name and Middle Initial(s)	*Last Name	*Suffix (eg, Jr, III)	Academic Degrees	Institution	Location (city, state/province, country)	Role or Contribution, eg, chair, principal investigator	Group (if more than 1 Group listed in the byline) and/or Subgroup (eg, Steering Committee)	
Brigette	Gomes		BScN, RN	Ottawa Civic Hospital	Ottawa, Ontario, Canada	Research Coordinator		
Lianne	Leclair		MScN	Ottawa Civic Hospital	Ottawa, Ontario, Canada	Research Coordinator		
Rebecca	Porteous		BScN, RN	Ottawa Civic Hospital	Ottawa, Ontario, Canada	Research Coordinator		
Shawna	Reddie		BSc	Ottawa Civic Hospital	Ottawa, Ontario, Canada	Research Coordinator		
Irene	Watpool		BScN, RN	Ottawa Civic Hospital	Ottawa, Ontario, Canada	Research Coordinator		
Marianne	Cox			Ottawa Civic Hospital	Ottawa, Ontario, Canada	Pharmacy		
Kathy	Fraser			Ottawa Civic Hospital	Ottawa, Ontario, Canada	Pharmacy		
Jennifer	Kuhn			Ottawa Civic Hospital	Ottawa, Ontario, Canada	Pharmacy		
Rob	Macleod			Ottawa Civic Hospital	Ottawa, Ontario, Canada	Pharmacy		
Susanne	Richard			Ottawa Civic Hospital	Ottawa, Ontario, Canada	Pharmacy		
Dawn	Rose			Ottawa Civic Hospital	Ottawa, Ontario, Canada	Pharmacy		
Sherry	Weir		RPhT	Ottawa Civic Hospital	Ottawa, Ontario, Canada	Pharmacy		
Lauralyn	McIntyre		MD	Ottawa General Hospital	Ottawa, Ontario, Canada	Site Investigator		
Pierre	Cardinal		MD	Ottawa General Hospital	Ottawa, Ontario, Canada	Site Co-Investigator		
Gianni	D`Egidio		MD	Ottawa General Hospital	Ottawa, Ontario, Canada	Site Co-Investigator		
Mike	Hartwick		MD	Ottawa General Hospital	Ottawa, Ontario, Canada	Site Co-Investigator		
Gwynne	Jones		MD	Ottawa General Hospital	Ottawa, Ontario, Canada	Site Co-Investigator		
John	Kim		MD	Ottawa General Hospital	Ottawa, Ontario, Canada	Site Co-Investigator		
Kwadwo	Kyeremanteng		MD	Ottawa General Hospital	Ottawa, Ontario, Canada	Site Co-Investigator		
Sherissa	Microys		MD	Ottawa General Hospital	Ottawa, Ontario, Canada	Site Co-Investigator		
Rakesh	Patel		MD	Ottawa General Hospital	Ottawa, Ontario, Canada	Site Co-Investigator		
Erin	Rosenberg		MD	Ottawa General Hospital	Ottawa, Ontario, Canada	Site Co-Investigator		
Aimee	Sarti		MD	Ottawa General Hospital	Ottawa, Ontario, Canada	Site Co-Investigator		
Andrew	Seely		MD	Ottawa General Hospital	Ottawa, Ontario, Canada	Site Co-Investigator		
Shelley	Acres		BScN, RN	Ottawa General Hospital	Ottawa, Ontario, Canada	Research Coordinator		
Sydney	Mietitis		BSc	Ottawa General Hospital	Ottawa, Ontario, Canada	Research Coordinator		
Kaitlyn	Montroy		MSc	Ottawa General Hospital	Ottawa, Ontario, Canada	Research Coordinator		
Rebecca	Porteous		BScN, RN	Ottawa General Hospital	Ottawa, Ontario, Canada	Research Coordinator		
Amanda	Van Beinum		MSc	Ottawa General Hospital	Ottawa, Ontario, Canada	Research Coordinator		
Irene	Watpool		BScN, RN	Ottawa General Hospital	Ottawa, Ontario, Canada	Research Coordinator		
Wendy	Aikens		RPhT	Ottawa General Hospital	Ottawa, Ontario, Canada	Pharmacy		
Anne-Marie	Dugal		RPhT	Ottawa General Hospital	Ottawa, Ontario, Canada	Pharmacy		
Susan	Fetzer		RPhT	Ottawa General Hospital	Ottawa, Ontario, Canada	Pharmacy		
Deborah	Cook		MD	St. Joseph`s Healthcare	Hamilton, Ontario, Canada	Site Investigator		
Erick	Duan		MD	St. Joseph`s Healthcare	Hamilton, Ontario, Canada	Site Co-Investigator		
Mark	Soth		MD	St. Joseph`s Healthcare	Hamilton, Ontario, Canada	Site Co-Investigator		
Waleed	Alhazanni		MD	St. Joseph`s Healthcare	Hamilton, Ontario, Canada	Site Co-Investigator		
France	Clarke		RRT	St. Joseph`s Healthcare	Hamilton, Ontario, Canada	Research Coordinator		
Mary	Copland		BSc	St. Joseph`s Healthcare	Hamilton, Ontario, Canada	Research Coordinator		
Neala	Hoad		RN	St. Joseph`s Healthcare	Hamilton, Ontario, Canada	Research Coordinator		
Marnie	Jakab		MD	St. Joseph`s Healthcare	Hamilton, Ontario, Canada	Research Coordinator		
Melissa	Sheers		MD	St. Joseph`s Healthcare	Hamilton, Ontario, Canada	Research Coordinator		
Alyson	Takaoka		MSc	St. Joseph`s Healthcare	Hamilton, Ontario, Canada	Research Coordinator		
Nicole	Zytaruk		RN	St. Joseph`s Healthcare	Hamilton, Ontario, Canada	Research Coordinator		
Christa	Connolly		BSc(Pharm)	St. Joseph`s Healthcare	Hamilton, Ontario, Canada	Pharmacy		
Denise	Davis		RPhT	St. Joseph`s Healthcare	Hamilton, Ontario, Canada	Pharmacy		
Catherine	Eaton		RPhT	St. Joseph`s Healthcare	Hamilton, Ontario, Canada	Pharmacy		
Tracy	Gallinas		BSc(Pharm), RPhT	St. Joseph`s Healthcare	Hamilton, Ontario, Canada	Pharmacy		
Jean	Lee-Yoo		BSc(Pharm), RPhT	St. Joseph`s Healthcare	Hamilton, Ontario, Canada	Pharmacy		
Connie	Lukinuk		BSc(Pharm), RPhT	St. Joseph`s Healthcare	Hamilton, Ontario, Canada	Pharmacy		
Leia	Musielak		PharmD	St. Joseph`s Healthcare	Hamilton, Ontario, Canada	Pharmacy		
Nancy	Pavunkovic		RPhT	St. Joseph`s Healthcare	Hamilton, Ontario, Canada	Pharmacy		
Joy	Pelayo		RPhT	St. Joseph`s Healthcare	Hamilton, Ontario, Canada	Pharmacy		
Kaitlyn	Phillips		RPhT	St. Joseph`s Healthcare	Hamilton, Ontario, Canada	Pharmacy		
Catherine	Pracsovics		RPhT	St. Joseph`s Healthcare	Hamilton, Ontario, Canada	Pharmacy		
Julia	Raimondo		PharmD, RPh	St. Joseph`s Healthcare	Hamilton, Ontario, Canada	Pharmacy		

*Indicates required information. Only first name, last name, and suffix will appear in PubMed.

*First Name and Middle Initial(s)	*Last Name	*Suffix (eg, Jr, III)	Academic Degrees	Institution	Location (city, state/province, country)	Role or Contribution, eg, chair, principal investigator	Group (if more than 1 Group listed in the byline) and/or Subgroup (eg, Steering Committee)	
Vida	Stankus		PharmD, RPh	St. Joseph`s Healthcare	Hamilton, Ontario, Canada	Pharmacy		
Christine	Wallace		BSc(Pharm), RPh	St. Joseph`s Healthcare	Hamilton, Ontario, Canada	Pharmacy		
Angela	Wright		PharmD, RPh	St. Joseph`s Healthcare	Hamilton, Ontario, Canada	Pharmacy		
Crystal	Young		RPhT	St. Joseph`s Healthcare	Hamilton, Ontario, Canada	Pharmacy		
Rob	Cirrone		MD, FRCPC	St. Joseph`s Health Center	Toronto, Ontario, Canada	Site Investigator		
Jennie	Johnstone		MD, PhD	St. Joseph`s Health Center	Toronto, Ontario, Canada	Site Co-Investigator		
Kanthi	Kavikondala		BSc, CCRP	St. Joseph`s Health Center	Toronto, Ontario, Canada	Research Coordinator		
Axelle	Pellerin	Pellerin	BA	St. Joseph`s Health Center	Toronto, Ontario, Canada	Research Coordinator		
Laura	Tomat		MSc, CCRP	St. Joseph`s Health Center	Toronto, Ontario, Canada	Research Coordinator		
Jeff	Carter		BSC(Pharm)	St. Joseph`s Health Center	Toronto, Ontario, Canada	Pharmacy		
Jiten	Jani		BSc(Pharm), ACRP	St. Joseph`s Health Center	Toronto, Ontario, Canada	Pharmacy		
Brendan	Yaets		MSc, ACRP	St. Joseph`s Health Center	Toronto, Ontario, Canada	Pharmacy		
John	Marshall		MD, MSc	St. Michael`s Hospital	Toronto, Ontario, Canada	Site Investigator		
Jan	Friedrich		MD, MSc	St. Michael`s Hospital	Toronto, Ontario, Canada	Site Co-Investigator		
Jennifer	Hodder		BScN	St. Michael`s Hospital	Toronto, Ontario, Canada	Research Coordinator		
Imrana	Khalid		MD	St. Michael`s Hospital	Toronto, Ontario, Canada	Research Coordinator		
Julie	Lee		BSc	St. Michael`s Hospital	Toronto, Ontario, Canada	Research Coordinator		
Yoon	Lee		BScN	St. Michael`s Hospital	Toronto, Ontario, Canada	Research Coordinator		
Kurtis	Salway		MSc	St. Michael`s Hospital	Toronto, Ontario, Canada	Research Coordinator		
Gyan	Sandhu		BScN	St. Michael`s Hospital	Toronto, Ontario, Canada	Research Coordinator		
Marlene	Santos		MD, MSc	St. Michael`s Hospital	Toronto, Ontario, Canada	Research Coordinator		
Orla	Smith		PhD	St. Michael`s Hospital	Toronto, Ontario, Canada	Research Coordinator		
Melissa	Wang		BScN	St. Michael`s Hospital	Toronto, Ontario, Canada	Research Coordinator		
Norman	Dewhurst		PharmD	St. Michael`s Hospital	Toronto, Ontario, Canada	Pharmacy		
Ann	Dowbenka		RPhT	St. Michael`s Hospital	Toronto, Ontario, Canada	Pharmacy		
Ann	Kosinski		RPhT	St. Michael`s Hospital	Toronto, Ontario, Canada	Pharmacy		
Laura	Parsons		RPhT	St. Michael`s Hospital	Toronto, Ontario, Canada	Pharmacy		
Johanna	Proceviat		BSc(Pharm)	St. Michael`s Hospital	Toronto, Ontario, Canada	Pharmacy		
Gitana	Ramonas		RPhT	St. Michael`s Hospital	Toronto, Ontario, Canada	Pharmacy		
Mae	Yuen		BSc(Pharm)	St. Michael`s Hospital	Toronto, Ontario, Canada	Pharmacy		
Neill	Adhikari		MD MSc	Sunnybrook Health Science Centre	Toronto, Ontario, Canada	Site Investigator		
Andre	Amaral		MD	Sunnybrook Health Science Centre	Toronto, Ontario, Canada	Site Co-Investigator		
Brian H	Cuthbertson		MD, FRCPC	Sunnybrook Health Science Centre	Toronto, Ontario, Canada	Site Co-Investigator		
Rob	Fowler		MD, MS	Sunnybrook Health Science Centre	Toronto, Ontario, Canada	Site Co-Investigator		
Damon	Scales		MD, PhD	Sunnybrook Health Science Centre	Toronto, Ontario, Canada	Site Co-Investigator		
Navjot	Kaur		MD	Sunnybrook Health Science Centre	Toronto, Ontario, Canada	Research Coordinator		
Nicole	Marinoff		RN	Sunnybrook Health Science Centre	Toronto, Ontario, Canada	Research Coordinator		
Adic	Perez		MD	Sunnybrook Health Science Centre	Toronto, Ontario, Canada	Research Coordinator		
Jane	Wang		MD	Sunnybrook Health Science Centre	Toronto, Ontario, Canada	Research Coordinator		
Katrina	Hatzifilalithis		RPhT	Sunnybrook Health Science Centre	Toronto, Ontario, Canada	Pharmacy		
John	Iazzetta		PharmD	Sunnybrook Health Science Centre	Toronto, Ontario, Canada	Pharmacy		
Chrys	Kolos		RPh	Sunnybrook Health Science Centre	Toronto, Ontario, Canada	Pharmacy		
Ingrid	Quinton		RPh	Sunnybrook Health Science Centre	Toronto, Ontario, Canada	Pharmacy		
Margaret	Herridge		MD	University Health Network - Toronto General Hospital	Toronto, Ontario, Canada	Site Investigator		
Eyal	Golan		MD, PhD	University Health Network - Toronto General Hospital	Toronto, Ontario, Canada	Site Co-Investigator		
John	Granton		MD	University Health Network - Toronto General Hospital	Toronto, Ontario, Canada	Site Co-Investigator		
Jaimie	Archer		HBSc	University Health Network - Toronto General Hospital	Toronto, Ontario, Canada	Research Coordinator		
Daniel	Chen		HBSc	University Health Network - Toronto General Hospital	Toronto, Ontario, Canada	Research Coordinator		
Brooke	Fraser		MD	University Health Network - Toronto General Hospital	Toronto, Ontario, Canada	Research Coordinator		
Cheryl	Geen-Smith		BScN	University Health Network - Toronto General Hospital	Toronto, Ontario, Canada	Research Coordinator		
Andrea	Matte		BSc, RRT	University Health Network - Toronto General Hospital	Toronto, Ontario, Canada	Research Coordinator		
Priscilla	Robles		PhD	University Health Network - Toronto General Hospital	Toronto, Ontario, Canada	Research Coordinator		
Cristian	Urrea		MD	University Health Network - Toronto General Hospital	Toronto, Ontario, Canada	Research Coordinator		
Jane	Ascroft		PharmD	University Health Network - Toronto General Hospital	Toronto, Ontario, Canada	Pharmacy		
Fatima	Haji		BSc(Pharm)	University Health Network - Toronto General Hospital	Toronto, Ontario, Canada	Pharmacy		
Jie	Ming		RPhT	University Health Network - Toronto General Hospital	Toronto, Ontario, Canada	Pharmacy		

Supplemental Online Content: Nonauthor Collaborators

*Indicates required information. Only first name, last name, and suffix will appear in PubMed.

***First Name and Middle Initial(s)**	***Last Name**	***Suffix (eg, Jr, III)**	Academic Degrees	Institution	Location (city, state/province, country)	Role or Contribution, eg, chair, principal investigator	Group (if more than 1 Group listed in the byline) and/or Subgroup (eg, Steering Committee)	
Matthew	Suen		RPhT	University Health Network - Toronto General Hospital	Toronto, Ontario, Canada	Pharmacy		
Muhammad	Walid		RPhT	University Health Network - Toronto General Hospital	Toronto, Ontario, Canada	Pharmacy		
Jill	Westlund		BSc(Pharm)	University Health Network - Toronto General Hospital	Toronto, Ontario, Canada	Pharmacy		
Margaret	Herridge		MD	University Health Network - Toronto WesternHospital	Toronto, Ontario, Canada	Site Investigator		
Alberto	Goffi		MD	University Health Network - Toronto WesternHospital	Toronto, Ontario, Canada	Site Co-Investigator		
Eyal	Golan		MD, PhD	University Health Network - Toronto WesternHospital	Toronto, Ontario, Canada	Site Co-Investigator		
John	Granton		MD	University Health Network - Toronto WesternHospital	Toronto, Ontario, Canada	Site Co-Investigator		
Victoria	McCredie		MD	University Health Network - Toronto WesternHospital	Toronto, Ontario, Canada	Site Co-Investigator		
Elizabeth	Wilcox		MD	University Health Network - Toronto WesternHospital	Toronto, Ontario, Canada	Site Co-Investigator		
Jaimie	Archer		HBSc	University Health Network - Toronto WesternHospital	Toronto, Ontario, Canada	Research Coordinator		
Daniel	Chen		HBSc	University Health Network - Toronto WesternHospital	Toronto, Ontario, Canada	Research Coordinator		
Paulina	Farias		BScN	University Health Network - Toronto WesternHospital	Toronto, Ontario, Canada	Research Coordinator		
Brooke	Fraser		MD	University Health Network - Toronto WesternHospital	Toronto, Ontario, Canada	Research Coordinator		
Cheryl	Geen-Smith		BScN	University Health Network - Toronto WesternHospital	Toronto, Ontario, Canada	Research Coordinator		
Barbara	Kosky		BSc	University Health Network - Toronto WesternHospital	Toronto, Ontario, Canada	Research Coordinator		
Andrea	Matte		BSc, RRT	University Health Network - Toronto WesternHospital	Toronto, Ontario, Canada	Research Coordinator		
Christina	Pugliese		MD	University Health Network - Toronto WesternHospital	Toronto, Ontario, Canada	Research Coordinator		
Priscilla	Robles		PhD	University Health Network - Toronto WesternHospital	Toronto, Ontario, Canada	Research Coordinator		
Lia	Stenyk		HBSc	University Health Network - Toronto WesternHospital	Toronto, Ontario, Canada	Research Coordinator		
Cristian	Urrea		MD	University Health Network - Toronto WesternHospital	Toronto, Ontario, Canada	Research Coordinator		
Karolina	Walczak		BA	University Health Network - Toronto WesternHospital	Toronto, Ontario, Canada	Research Coordinator		
Kyung	Ae			University Health Network - Toronto WesternHospital	Toronto, Ontario, Canada	Pharmacy		
Jane	Ascroft		PharmD	University Health Network - Toronto WesternHospital	Toronto, Ontario, Canada	Pharmacy		
Fatima	Haji		BSc(Pharm)	University Health Network - Toronto WesternHospital	Toronto, Ontario, Canada	Pharmacy		
Rajvinder	Kaur		RPhT	University Health Network - Toronto WesternHospital	Toronto, Ontario, Canada	Pharmacy		
Jane	Lui		BSc(Pharm)	University Health Network - Toronto WesternHospital	Toronto, Ontario, Canada	Pharmacy		
Sophia	Mateo		RPhT	University Health Network - Toronto WesternHospital	Toronto, Ontario, Canada	Pharmacy		
Nga	Pham		RPhT	University Health Network - Toronto WesternHospital	Toronto, Ontario, Canada	Pharmacy		
Tam	Pham		RPhT	University Health Network - Toronto WesternHospital	Toronto, Ontario, Canada	Pharmacy		
Matthew	Suen		RPhT	University Health Network - Toronto WesternHospital	Toronto, Ontario, Canada	Pharmacy		
Sebastien	Trop		MD, PhD	William Osler Hospital, McKenzie Health	Brampton, Ontario, Canada	Site Investigator		
Alexandra	Binnie		MD, DPhil	William Osler Hospital, McKenzie Health	Brampton, Ontario, Canada	Site Co-Investigator		
Ronald	Heslegrave		MD	William Osler Hospital, McKenzie Health	Brampton, Ontario, Canada	Site Co-Investigator		
Zaynab	Panchbhaya		BScN	William Osler Hospital, McKenzie Health	Brampton, Ontario, Canada	Research Coordinator		
Kim	Sharman		RPhT	William Osler Hospital, McKenzie Health	Brampton, Ontario, Canada	Pharmacy		
Rakhi	Goel		BSc(Pharm)	William Osler Hospital, McKenzie Health	Brampton, Ontario, Canada	Pharmacy		
Kim	Kozluk		RPhT	William Osler Hospital, McKenzie Health	Brampton, Ontario, Canada	Pharmacy		
Julianne	Labelle		MHA	William Osler Hospital, McKenzie Health	Brampton, Ontario, Canada	Pharmacy		
Hina	Marsonia		PharmD	William Osler Hospital, McKenzie Health	Brampton, Ontario, Canada	Pharmacy		
Cecillia	Scott		RPhT	William Osler Hospital, McKenzie Health	Brampton, Ontario, Canada	Pharmacy		
Martin	Girard		MD, MSc	Centre hospitalier de Université de Montréal	Montréal, Quebec, Canada	Site Investigator		
Pierrre	Aslanian		MD	Centre hospitalier de Université de Montréal	Montréal, Quebec, Canada	Site Co-Investigator		
Sylvain	Belisle		MD	Centre hospitalier de Université de Montréal	Montréal, Quebec, Canada	Site Co-Investigator		
François-Martin	Carrier		MD, MSc	Centre hospitalier de Université de Montréal	Montréal, Quebec, Canada	Site Co-Investigator		
Michaël	Chassé		MD, PhD	Centre hospitalier de Université de Montréal	Montréal, Quebec, Canada	Site Co-Investigator		
André	Denault		MD, PhD	Centre hospitalier de Université de Montréal	Montréal, Quebec, Canada	Site Co-Investigator		
Jean-Gilles	Guimond		MD	Centre hospitalier de Université de Montréal	Montréal, Quebec, Canada	Site Co-Investigator		
Antoine	Halwagi		MD	Centre hospitalier de Université de Montréal	Montréal, Quebec, Canada	Site Co-Investigator		
Paul	Hébert		MD, MSc	Centre hospitalier de Université de Montréal	Montréal, Quebec, Canada	Site Co-Investigator		
Christopher	Kolan		MD	Centre hospitalier de Université de Montréal	Montréal, Quebec, Canada	Site Co-Investigator		
Jordi	Mancebo		MD, PhD	Centre hospitalier de Université de Montréal	Montréal, Quebec, Canada	Site Co-Investigator		
Nicholas	Robillard		MD	Centre hospitalier de Université de Montréal	Montréal, Quebec, Canada	Site Co-Investigator		
Fatna	Benettaib		MSc, BSc	Centre hospitalier de Université de Montréal	Montréal, Quebec, Canada	Research Coordinator		
Dounia	Boumahni		BScN, BSc	Centre hospitalier de Université de Montréal	Montréal, Quebec, Canada	Research Coordinator		
Casey	Bourdeau Caporuscio		BSc	Centre hospitalier de Université de Montréal	Montréal, Quebec, Canada	Research Coordinator		
Marie-Ève	Cantin		MSc	Centre hospitalier de Université de Montréal	Montréal, Quebec, Canada	Research Coordinator		

*Indicates required information. Only first name, last name, and suffix will appear in PubMed.

*First Name and Middle Initial(s)	*Last Name	*Suffix (eg, Jr, III)	Academic Degrees	Institution	Location (city, state/province, country)	Role or Contribution, eg, chair, principal investigator	Group (if more than 1 Group listed in the byline) and/or Subgroup (eg, Steering Committee)	
Virginy	Côté-Gravel		BSc	Centre hospitalier de Université de Montréal	Montréal, Quebec, Canada	Research Coordinator		
Ali	Ghamraoui		BSc	Centre hospitalier de Université de Montréal	Montréal, Quebec, Canada	Research Coordinator		
Martine	Lebrasseur		RN	Centre hospitalier de Université de Montréal	Montréal, Quebec, Canada	Research Coordinator		
Lancelot	Legendre Courville		BSc	Centre hospitalier de Université de Montréal	Montréal, Quebec, Canada	Research Coordinator		
Stéphanie	Lorio		DEC	Centre hospitalier de Université de Montréal	Montréal, Quebec, Canada	Research Coordinator		
Maria	Trinidad Madrid		AEC	Centre hospitalier de Université de Montréal	Montréal, Quebec, Canada	Research Coordinator		
Nicole	Poitras		DESS	Centre hospitalier de Université de Montréal	Montréal, Quebec, Canada	Research Coordinator		
Romain	Rigal		PharmD	Centre hospitalier de Université de Montréal	Montréal, Quebec, Canada	Research Coordinator		
Maya	Salame		BSc	Centre hospitalier de Université de Montréal	Montréal, Quebec, Canada	Research Coordinator		
Valérie	Tran		MSc	Centre hospitalier de Université de Montréal	Montréal, Quebec, Canada	Research Coordinator		
Katie	Bacon		DEP	Centre hospitalier de Université de Montréal	Montréal, Quebec, Canada	Pharmacy		
Nathalie	Boudreau		MSc, BSC(Pharm)	Centre hospitalier de Université de Montréal	Montréal, Quebec, Canada	Pharmacy		
Cecilia	Carvajal			Centre hospitalier de Université de Montréal	Montréal, Quebec, Canada	Pharmacy		
Lyne	Gauthier		MSc, BSc(Pharm)	Centre hospitalier de Université de Montréal	Montréal, Quebec, Canada	Pharmacy		
Julie	Gendron		BSc	Centre hospitalier de Université de Montréal	Montréal, Quebec, Canada	Pharmacy		
Karine	Jean			Centre hospitalier de Université de Montréal	Montréal, Quebec, Canada	Pharmacy		
Louise	Laforest		MSc, BSc(Pharm)	Centre hospitalier de Université de Montréal	Montréal, Quebec, Canada	Pharmacy		
Antonietta	Lembo			Centre hospitalier de Université de Montréal	Montréal, Quebec, Canada	Pharmacy		
Sothun	Lim		BSc(Pharm)	Centre hospitalier de Université de Montréal	Montréal, Quebec, Canada	Pharmacy		
Jennifer	Morrissette		DEP	Centre hospitalier de Université de Montréal	Montréal, Quebec, Canada	Pharmacy		
France	Pagé			Centre hospitalier de Université de Montréal	Montréal, Quebec, Canada	Pharmacy		
Lucie	Pelletier			Centre hospitalier de Université de Montréal	Montréal, Quebec, Canada	Pharmacy		
Marie-Christine	Rodrigue		MSc, BSc(Pharm)	Centre hospitalier de Université de Montréal	Montréal, Quebec, Canada	Pharmacy		
Patrick	Archambault		MD, MSc	Centre intégré de santé et de services sociaux de Chaudièı	Lévis, Quebec, Canada	Site Investigator		
Jean-François	Bellemare		MD	Centre intégré de santé et de services sociaux de Chaudièı	Lévis, Quebec, Canada	Site Co-Investigator		
Simon	Bordeleau		MD	Centre intégré de santé et de services sociaux de Chaudièı	Lévis, Quebec, Canada	Site Co-Investigator		
Christine	Drouin		MD	Centre intégré de santé et de services sociaux de Chaudièı	Lévis, Quebec, Canada	Site Co-Investigator		
Benoit	Duhaime		MD	Centre intégré de santé et de services sociaux de Chaudièı	Lévis, Quebec, Canada	Site Co-Investigator		
Ann	Laberge		MD, MBA	Centre intégré de santé et de services sociaux de Chaudièı	Lévis, Quebec, Canada	Site Co-Investigator		
Philippe	Lechance		MD, MSc	Centre intégré de santé et de services sociaux de Chaudièı	Lévis, Quebec, Canada	Site Co-Investigator		
Estel	Deblois		BSc Inf	Centre intégré de santé et de services sociaux de Chaudièı	Lévis, Quebec, Canada	Research Coordinator		
Maude	Dionne		MSc	Centre intégré de santé et de services sociaux de Chaudièı	Lévis, Quebec, Canada	Research Coordinator		
Lise	Lavoie		DEC (diplôme d'études	Centre intégré de santé et de services sociaux de Chaudièı	Lévis, Quebec, Canada	Research Coordinator		
Isabelle	Michel		BSc Inf	Centre intégré de santé et de services sociaux de Chaudièı	Lévis, Quebec, Canada	Research Coordinator		
Alexandre	Pépin		BSc	Centre intégré de santé et de services sociaux de Chaudièı	Lévis, Quebec, Canada	Research Coordinator		
Sandrine	Poulin		BA	Centre intégré de santé et de services sociaux de Chaudièı	Lévis, Quebec, Canada	Research Coordinator		
Sarah	Anctil		MSc	Centre intégré de santé et de services sociaux de Chaudièı	Lévis, Quebec, Canada	Pharmacy		
Louis-Étienne	Marchand		MSc	Centre intégré de santé et de services sociaux de Chaudièı	Lévis, Quebec, Canada	Pharmacy		
Robin	Roy		BSc	Centre intégré de santé et de services sociaux de Chaudièı	Lévis, Quebec, Canada	Intenive Care Unit		
François	Lamontagne		MD	Centre intégré universitaire de santé et de services sociau	Sherbrooke, Quebec, Canada	Site Investigator		
Charles	St-Arnaud		MD	Centre intégré universitaire de santé et de services sociau	Sherbrooke, Quebec, Canada	Site Co-Investigator		
Frédérick	D'Aragon		MD	Centre intégré universitaire de santé et de services sociau	Sherbrooke, Quebec, Canada	Site Co-Investigator		
Hector	Quiroz		MD	Centre intégré universitaire de santé et de services sociau	Sherbrooke, Quebec, Canada	Site Co-Investigator		
Dominique	Bérard		MD	Centre intégré universitaire de santé et de services sociau	Sherbrooke, Quebec, Canada	Site Co-Investigator		
Marc-Andre	Léclair		MD	Centre intégré universitaire de santé et de services sociau	Sherbrooke, Quebec, Canada	Site Co-Investigator		
Virginie	Bolduc		RN	Centre intégré universitaire de santé et de services sociau	Sherbrooke, Quebec, Canada	Research Coordinator		
Elaine	Carbonneau		RN	Centre intégré universitaire de santé et de services sociau	Sherbrooke, Quebec, Canada	Research Coordinator		
Joannie	Marchand			Centre intégré universitaire de santé et de services sociau	Sherbrooke, Quebec, Canada	Research Coordinator		
Marie-Hélène	Masse		MSc	Centre intégré universitaire de santé et de services sociau	Sherbrooke, Quebec, Canada	Research Coordinator		
Sylvie	Cloutier		MSc	Centre intégré universitaire de santé et de services sociau	Sherbrooke, Quebec, Canada	Pharmacy		
Marianne	Guay		MSc	Centre intégré universitaire de santé et de services sociau	Sherbrooke, Quebec, Canada	Pharmacy		
Line	Morin			Centre intégré universitaire de santé et de services sociau	Sherbrooke, Quebec, Canada	Pharmacy		
Jessie	Nicolson			Centre intégré universitaire de santé et de services sociau	Sherbrooke, Quebec, Canada	Pharmacy		
Isabelle	Paquette			Centre intégré universitaire de santé et de services sociau	Sherbrooke, Quebec, Canada	Pharmacy		
France	Théberge			Centre intégré universitaire de santé et de services sociau	Sherbrooke, Quebec, Canada	Pharmacy		
François	Lamontagne		MD	Centre intégré universitaire de santé et de services sociau	Sherbrooke, Quebec, Canada	Site Investigator		

*Indicates required information. Only first name, last name, and suffix will appear in PubMed.

*First Name and Middle Initial(s)	*Last Name	*Suffix (eg, Jr, III)	Academic Degrees	Institution	Location (city, state/province, country)	Role or Contribution, eg, chair, principal investigator	Group (if more than 1 Group listed in the byline) and/or Subgroup (eg, Steering Committee)	
Charles	St-Arnaud		MD	Centre intégré universitaire de santé et de services sociau	Sherbrooke, Quebec, Canada	Site Co-Investigator		
Frédérick	D'Aragon		MD	Centre intégré universitaire de santé et de services sociau	Sherbrooke, Quebec, Canada	Site Co-Investigator		
Hector	Quiroz		MD	Centre intégré universitaire de santé et de services sociau	Sherbrooke, Quebec, Canada	Site Co-Investigator		
Dominique	Bérard		MD	Centre intégré universitaire de santé et de services sociau	Sherbrooke, Quebec, Canada	Site Co-Investigator		
Marc-Andre	Léclair		MD	Centre intégré universitaire de santé et de services sociau	Sherbrooke, Quebec, Canada	Site Co-Investigator		
Virginie	Bolduc		RN	Centre intégré universitaire de santé et de services sociau	Sherbrooke, Quebec, Canada	Research Coordinator		
Elaine	Carbonneau		RN	Centre intégré universitaire de santé et de services sociau	Sherbrooke, Quebec, Canada	Research Coordinator		
Joannie	Marchand			Centre intégré universitaire de santé et de services sociau	Sherbrooke, Quebec, Canada	Research Coordinator		
Marie-Hélène	Masse		MSc	Centre intégré universitaire de santé et de services sociau	Sherbrooke, Quebec, Canada	Research Coordinator		
Sylvie	Cloutier		MSc	Centre intégré universitaire de santé et de services sociau	Sherbrooke, Quebec, Canada	Pharmacy		
Marianne	Guay		MSc	Centre intégré universitaire de santé et de services sociau	Sherbrooke, Quebec, Canada	Pharmacy		
Line	Morin			Centre intégré universitaire de santé et de services sociau	Sherbrooke, Quebec, Canada	Pharmacy		
Jessie	Nicolson			Centre intégré universitaire de santé et de services sociau	Sherbrooke, Quebec, Canada	Pharmacy		
Isabelle	Paquette			Centre intégré universitaire de santé et de services sociau	Sherbrooke, Quebec, Canada	Pharmacy		
France	Théberge			Centre intégré universitaire de santé et de services sociau	Sherbrooke, Quebec, Canada	Pharmacy		
Emmanuel	Charbonney		MD, PhD	Centre intégré universitaire de santé et de services sociau	Montréal, Quebec, Canada	Site Investigator		
Yoan	Lamarche		MD	Centre intégré universitaire de santé et de services sociau	Montréal, Quebec, Canada	Site Co-Investigator		
Soazig	Leguillan		MD	Centre intégré universitaire de santé et de services sociau	Montréal, Quebec, Canada	Site Co-Investigator		
Karim	Serri		MD	Centre intégré universitaire de santé et de services sociau	Montréal, Quebec, Canada	Site Co-Investigator		
Colin	Verdant		MD	Centre intégré universitaire de santé et de services sociau	Montréal, Quebec, Canada	Site Co-Investigator		
Yanick	Beaulieu		MD	Centre intégré universitaire de santé et de services sociau	Montréal, Quebec, Canada	Site Co-Investigator		
Patrick	Bellemare		MD	Centre intégré universitaire de santé et de services sociau	Montréal, Quebec, Canada	Site Co-Investigator		
Philippe	Rico		MD	Centre intégré universitaire de santé et de services sociau	Montréal, Quebec, Canada	Site Co-Investigator		
Francis	Bernard		MD	Centre intégré universitaire de santé et de services sociau	Montréal, Quebec, Canada	Site Co-Investigator		
Marc	Giasson		MD	Centre intégré universitaire de santé et de services sociau	Montréal, Quebec, Canada	Site Co-Investigator		
Véronique	Brunette		MD	Centre intégré universitaire de santé et de services sociau	Montréal, Quebec, Canada	Site Co-Investigator		
Alexandros	Cavayas		MD, MSc	Centre intégré universitaire de santé et de services sociau	Montréal, Quebec, Canada	Site Co-Investigator		
Émilie	Lévesque		MD	Centre intégré universitaire de santé et de services sociau	Montréal, Quebec, Canada	Site Co-Investigator		
Halina	Labikova		BSc	Centre intégré universitaire de santé et de services sociau	Montréal, Quebec, Canada	Research Coordinator		
Julia	Lainer Palacios		MSc	Centre intégré universitaire de santé et de services sociau	Montréal, Quebec, Canada	Research Coordinator		
Marie-Ève	Langlois		MSc	Centre intégré universitaire de santé et de services sociau	Montréal, Quebec, Canada	Research Coordinator		
Virginie	Williams		PhD	Centre intégré universitaire de santé et de services sociau	Montréal, Quebec, Canada	Research Coordinator		
Thuy Anh	Nguyen		MSc, B.Pharm	Centre intégré universitaire de santé et de services sociau	Montréal, Quebec, Canada	Pharmacy		
Valérie	Phaneuf		B.Pharm, MSc	Centre intégré universitaire de santé et de services sociau	Montréal, Quebec, Canada	Pharmacy		
David	Williamson		PhD, B.Pharm	Centre intégré universitaire de santé et de services sociau	Montréal, Quebec, Canada	Pharmacy		
François	Marquis		MD, MA	Centre intégré universitaire de santé et de services sociau	Montréal, Quebec, Canada	Site Investigator		
Han	Tin Wang		MD, MSc	Centre intégré universitaire de santé et de services sociau	Montréal, Quebec, Canada	Site Co-Investigator		
Francis	Toupin		MD, MMEd, FRCPC	Centre intégré universitaire de santé et de services sociau	Montréal, Quebec, Canada	Site Co-Investigator		
Stephane	Ahern		MD, MA, PhD	Centre intégré universitaire de santé et de services sociau	Montréal, Quebec, Canada	Site Co-Investigator		
Brian	Laufer		MD	Centre intégré universitaire de santé et de services sociau	Montréal, Quebec, Canada	Site Co-Investigator		
Marc	Brosseau		MD	Centre intégré universitaire de santé et de services sociau	Montréal, Quebec, Canada	Site Co-Investigator		
Pauline	Dul			Centre intégré universitaire de santé et de services sociau	Montréal, Quebec, Canada	Research Coordinator		
Johanne	Harvey		RN	Centre intégré universitaire de santé et de services sociau	Montréal, Quebec, Canada	Research Coordinator		
Lotthida	Inthanavong		MSc	Centre intégré universitaire de santé et de services sociau	Montréal, Quebec, Canada	Research Coordinator		
Danae	Tassy		PhD, RN	Centre intégré universitaire de santé et de services sociau	Montréal, Quebec, Canada	Research Coordinator		
Helen	Assayag		MSc(Pharm)	Centre intégré universitaire de santé et de services sociau	Montréal, Quebec, Canada	Pharmacy		
Maude	Bachand		MSc(Pharm)	Centre intégré universitaire de santé et de services sociau	Montréal, Quebec, Canada	Pharmacy		
Marysa	Betournay		PA	Centre intégré universitaire de santé et de services sociau	Montréal, Quebec, Canada	Pharmacy		
Karine	Daoust		MSc(Pharm)	Centre intégré universitaire de santé et de services sociau	Montréal, Quebec, Canada	Pharmacy		
Kristine	Goyette		MSc(Pharm)	Centre intégré universitaire de santé et de services sociau	Montréal, Quebec, Canada	Pharmacy		
Ariane	Lessard		MSc(Pharm)	Centre intégré universitaire de santé et de services sociau	Montréal, Quebec, Canada	Pharmacy		
Marceline	Quach		MSc(Pharm)	Centre intégré universitaire de santé et de services sociau	Montréal, Quebec, Canada	Pharmacy		
François	Lauzier		MD, MSc, FRCPC	CHU de Québec-Université Laval (Hôpital de l'Enfant-Jésus	Québec City, Quebec, Canada	Site Investigator		
Alexis	Turgeon		MD, MSc, FRCPC	CHU de Québec-Université Laval (Hôpital de l'Enfant-Jésus	Québec City, Quebec, Canada	Site Co-Investigator		
Danny	Barriault		MD	CHU de Québec-Université Laval (Hôpital de l'Enfant-Jésus	Québec City, Quebec, Canada	Research Coordinator		
David	Bellemare		MSc, RN	CHU de Québec-Université Laval (Hôpital de l'Enfant-Jésus	Québec City, Quebec, Canada	Research Coordinator		
Anick	Boivin		BSc, RN	CHU de Québec-Université Laval (Hôpital de l'Enfant-Jésus	Québec City, Quebec, Canada	Research Coordinator		

Supplemental Online Content: Nonauthor Collaborators

*Indicates required information. Only first name, last name, and suffix will appear in PubMed.

*First Name and Middle Initial(s)	*Last Name	*Suffix (eg, Jr, III)	Academic Degrees	Institution	Location (city, state/province, country)	Role or Contribution, eg, chair, principal investigator	Group (if more than 1 Group listed in the byline) and/or Subgroup (eg, Steering Committee)	
Sarah-Judith	Breton		MSc, RN	CHU de Québec-Université Laval (Hôpital de l'Enfant-Jésus	Québec City, Quebec, Canada	Research Coordinator		
Ève	Cloutier			CHU de Québec-Université Laval (Hôpital de l'Enfant-Jésus	Québec City, Quebec, Canada	Research Coordinator		
Marjorie	Daigle			CHU de Québec-Université Laval (Hôpital de l'Enfant-Jésus	Québec City, Quebec, Canada	Research Coordinator		
Charles	Delisle-Thibeault		BSc RN	CHU de Québec-Université Laval (Hôpital de l'Enfant-Jésus	Québec City, Quebec, Canada	Research Coordinator		
Panagiota	Giannakouros		BSc	CHU de Québec-Université Laval (Hôpital de l'Enfant-Jésus	Québec City, Quebec, Canada	Research Coordinator		
Stéphanie	Grenier		BSc, RN	CHU de Québec-Université Laval (Hôpital de l'Enfant-Jésus	Québec City, Quebec, Canada	Research Coordinator		
Gabrielle	Guilbault			CHU de Québec-Université Laval (Hôpital de l'Enfant-Jésus	Québec City, Quebec, Canada	Research Coordinator		
Caroline	Léger		PhD	CHU de Québec-Université Laval (Hôpital de l'Enfant-Jésus	Québec City, Quebec, Canada	Research Coordinator		
Catherine	Ouellet		MSc	CHU de Québec-Université Laval (Hôpital de l'Enfant-Jésus	Québec City, Quebec, Canada	Research Coordinator		
Marie-Claude	Trembley		BSc, Rn	CHU de Québec-Université Laval (Hôpital de l'Enfant-Jésus	Québec City, Quebec, Canada	Research Coordinator		
Julie	Gaudreau		MSc, BSc(Pharm)	CHU de Québec-Université Laval (Hôpital de l'Enfant-Jésus	Québec City, Quebec, Canada	Pharmacy		
Claire	Grégoire		MSc, BSc(Pharm)	CHU de Québec-Université Laval (Hôpital de l'Enfant-Jésus	Québec City, Quebec, Canada	Pharmacy		
Véronique	Labbé		MSc, BSc(Pharm)	CHU de Québec-Université Laval (Hôpital de l'Enfant-Jésus	Québec City, Quebec, Canada	Pharmacy		
Ariane	Laprise-Rochette		MSc, BSc(Pharm)	CHU de Québec-Université Laval (Hôpital de l'Enfant-Jésus	Québec City, Quebec, Canada	Pharmacy		
Caroline	Ouellet		MSc	CHU de Québec-Université Laval (Hôpital de l'Enfant-Jésus	Québec City, Quebec, Canada	Pharmacy		
Mélanie	Samson		BSc(Pharm)	CHU de Québec-Université Laval (Hôpital de l'Enfant-Jésus	Québec City, Quebec, Canada	Pharmacy		
Marie-David	Simoneau		MSc, BSc(Pharm)	CHU de Québec-Université Laval (Hôpital de l'Enfant-Jésus	Québec City, Quebec, Canada	Pharmacy		
Virginie	Tourcotte		MSc, BSc(Pharm)	CHU de Québec-Université Laval (Hôpital de l'Enfant-Jésus	Québec City, Quebec, Canada	Pharmacy		
Tuong-Vi	Tran		MSc, BSc(Pharm)	CHU de Québec-Université Laval (Hôpital de l'Enfant-Jésus	Québec City, Quebec, Canada	Pharmacy		
François	Lellouche		MD, PhD	Institut Universitaire de Cardiologie et de Pneumologie de	Québec City, Quebec, Canada	Site Investigator		
Ying	Tung Sia		MD, FRCPC	Institut Universitaire de Cardiologie et de Pneumologie de	Québec City, Quebec, Canada	Site Co-Investigator		
Mathieu	Simon		MD, FRCPC	Institut Universitaire de Cardiologie et de Pneumologie de	Québec City, Quebec, Canada	Site Co-Investigator		
Pierre-Alexandre	Bouchard		RRT	Institut Universitaire de Cardiologie et de Pneumologie de	Québec City, Quebec, Canada	Research Coordinator		
Patricia	Lizotte		RN	Institut Universitaire de Cardiologie et de Pneumologie de	Québec City, Quebec, Canada	Research Coordinator		
Nathalie	Chateauvert		BSc(Pharm)	Institut Universitaire de Cardiologie et de Pneumologie de	Québec City, Quebec, Canada	Pharmacy		
Thérèse	Grenier			Institut Universitaire de Cardiologie et de Pneumologie de	Québec City, Quebec, Canada	Pharmacy		
Kosar	Khwaja		MD, FRCPC	McGill University Health Centre (Montréal General Hospit	Montréal, Quebec, Canada	Site Investigator		
Dan	Deckelbaum		MD, FRCPC	McGill University Health Centre (Montréal General Hospit	Montréal, Quebec, Canada	Site Co-Investigator		
Jeremy	Grushka		MD, FRCPC	McGill University Health Centre (Montréal General Hospit	Montréal, Quebec, Canada	Site Co-Investigator		
Ash	Gursahaney		MD, FRCPC	McGill University Health Centre (Montréal General Hospit	Montréal, Quebec, Canada	Site Co-Investigator		
David	Hornstein		MD, FRCPC	McGill University Health Centre (Montréal General Hospit	Montréal, Quebec, Canada	Site Co-Investigator		
Dev	Jayaraman		MD, FRCPC	McGill University Health Centre (Montréal General Hospit	Montréal, Quebec, Canada	Site Co-Investigator		
Tarek	Razek		MD, FRCPC	McGill University Health Centre (Montréal General Hospit	Montréal, Quebec, Canada	Site Co-Investigator		
Robert	Salasidis		MD, FRCPC	McGill University Health Centre (Montréal General Hospit	Montréal, Quebec, Canada	Site Co-Investigator		
Patrizia	Zanelli		MD, FRCPC	McGill University Health Centre (Montréal General Hospit	Montréal, Quebec, Canada	Site Co-Investigator		
Norine	Alam		MD	McGill University Health Centre (Montréal General Hospit	Montréal, Quebec, Canada	Research Coordinator		
Laura	Garcia		RN	McGill University Health Centre (Montréal General Hospit	Montréal, Quebec, Canada	Research Coordinator		
Tonia	Doerksen		PhD, BPharm	McGill University Health Centre (Montréal General Hospit	Montréal, Quebec, Canada	Pharmacy		
Ariane	Lessard		MSc, BPharm	McGill University Health Centre (Montréal General Hospit	Montréal, Quebec, Canada	Pharmacy		
Gilbert	Matte		PhD, MSc, BPharm	McGill University Health Centre (Montréal General Hospit	Montréal, Quebec, Canada	Pharmacy		
Marie-France	Robert		MSc	McGill University Health Centre (Montréal General Hospit	Montréal, Quebec, Canada	Pharmacy		
Arnold S	Kristof		MD, FRCPC	McGill University Health Centre (Royal Victoria Hospital, G	Montréal, Quebec, Canada	Site Investigator		
Peter	Goldberg		MD, FRCPC	McGill University Health Centre (Royal Victoria Hospital, G	Montréal, Quebec, Canada	Site Co-Investigator		
Roupen	Hatzakorzian		MD, FRCPC	McGill University Health Centre (Royal Victoria Hospital, G	Montréal, Quebec, Canada	Site Co-Investigator		
Sheldon	Magder		MD, FRCPC	McGill University Health Centre (Royal Victoria Hospital, G	Montréal, Quebec, Canada	Site Co-Investigator		
Jason	Shahin		MD, FRCPC	McGill University Health Centre (Royal Victoria Hospital, G	Montréal, Quebec, Canada	Site Co-Investigator		
Salman	Qureshi		MD, FRCPC	McGill University Health Centre (Royal Victoria Hospital, G	Montréal, Quebec, Canada	Site Co-Investigator		
Josie	Campisi		MSc	McGill University Health Centre (Royal Victoria Hospital, G	Montréal, Quebec, Canada	Research Coordinator		
Vasilica	Botan		DEP	McGill University Health Centre (Royal Victoria Hospital, G	Montréal, Quebec, Canada	Pharmacy		
Anissa	Capilnean		MSc, BPharm	McGill University Health Centre (Royal Victoria Hospital, G	Montréal, Quebec, Canada	Pharmacy		
Alyssa	Corey		DEP	McGill University Health Centre (Royal Victoria Hospital, G	Montréal, Quebec, Canada	Pharmacy		
Annick	Gagné		DEP	McGill University Health Centre (Royal Victoria Hospital, G	Montréal, Quebec, Canada	Pharmacy		
Jasmine	Mian		BPharm, DPH	McGill University Health Centre (Royal Victoria Hospital, G	Montréal, Quebec, Canada	Pharmacy		
Kathleen	Normandin		MSc, BPharm	McGill University Health Centre (Royal Victoria Hospital, G	Montréal, Quebec, Canada	Pharmacy		
Osama	Loubani		MD, FRCPC	Nova Scotia Health Authority QEII	Halifax, Nova Scotia, Canada	Site Investigator		
Rick	Hall		MD, FRCPC	Nova Scotia Health Authority QEII	Halifax, Nova Scotia, Canada	Site Co-Investigator		

Supplemental Online Content: Nonauthor Collaborators

*Indicates required information. Only first name, last name, and suffix will appear in PubMed.

*First Name and Middle Initial(s)	*Last Name	*Suffix (eg, Jr, III)	Academic Degrees	Institution	Location (city, state/province, country)	Role or Contribution, eg, chair, principal investigator	Group (if more than 1 Group listed in the byline) and/or Subgroup (eg, Steering Committee)	
Robert	Green		MD, FRCPC	Nova Scotia Health Authority QEII	Halifax, Nova Scotia, Canada	Site Co-Investigator		
Diana	Gillis		BScN	Nova Scotia Health Authority QEII	Halifax, Nova Scotia, Canada	Research Coordinator		
Lisa	Julien		BScN	Nova Scotia Health Authority QEII	Halifax, Nova Scotia, Canada	Research Coordinator		
Laura Lee	Magennis		BN	Nova Scotia Health Authority QEII	Halifax, Nova Scotia, Canada	Research Coordinator		
Tamara	Mitterer		BScN, BSc	Nova Scotia Health Authority QEII	Halifax, Nova Scotia, Canada	Research Coordinator		
Joanna	Arsenault		CCRP, PhT	Nova Scotia Health Authority QEII	Halifax, Nova Scotia, Canada	Pharmacy		
Kim	Bruce-Payne		RPhT	Nova Scotia Health Authority QEII	Halifax, Nova Scotia, Canada	Pharmacy		
Patti	Gallant		CCRP, PhT	Nova Scotia Health Authority QEII	Halifax, Nova Scotia, Canada	Pharmacy		
Rodrigo	Cartin-Ceba		MD	Mayo Clinic, Rochester	Rochester, Minnisota, United States of America	Site Investigator		
Richard	Oeckler		MD, PhD	Mayo Clinic, Rochester	Rochester, Minnisota, United States of America	Site Co-Investigator		
Brenda	Anderson		BScN	Mayo Clinic, Rochester	Rochester, Minnisota, United States of America	Research Coordinator		
Lavonne	Liedl		RRT, LRT	Mayo Clinic, Rochester	Rochester, Minnisota, United States of America	Research Coordinator		
Laurie	Meade		RN, AD	Mayo Clinic, Rochester	Rochester, Minnisota, United States of America	Research Coordinator		
Sueanne	Weist		BScN	Mayo Clinic, Rochester	Rochester, Minnisota, United States of America	Research Coordinator		
Anna	Bartoo		PharmD, RPh	Mayo Clinic, Rochester	Rochester, Minnisota, United States of America	Pharmacy		
Debbie	Bauer		PharmD, RPh	Mayo Clinic, Rochester	Rochester, Minnisota, United States of America	Pharmacy		
Vince	Brickley		PharmD, RPh	Mayo Clinic, Rochester	Rochester, Minnisota, United States of America	Pharmacy		
Shaun	Bridges		RPh	Mayo Clinic, Rochester	Rochester, Minnisota, United States of America	Pharmacy		
Greg	Brunn		DRPh	Mayo Clinic, Rochester	Rochester, Minnisota, United States of America	Pharmacy		
Jennifer	Eickstaedt		DRPh	Mayo Clinic, Rochester	Rochester, Minnisota, United States of America	Pharmacy		
Jill	Bergerson		PharmD, RPh	Mayo Clinic, Rochester	Rochester, Minnisota, United States of America	Pharmacy		
Sandy	Showalter		RPh	Mayo Clinic, Rochester	Rochester, Minnisota, United States of America	Pharmacy		
Erin	Stern		PharmD, RPh	Mayo Clinic, Rochester	Rochester, Minnisota, United States of America	Pharmacy		
Melissa	Manley		PharmD, RPh	Mayo Clinic, Rochester	Rochester, Minnisota, United States of America	Pharmacy		
Robert	Taylor		MD	St John’s Mercy Medical Center	St Louis, Missouri, United States of America	Site Investigator		
Margaret	Cryton		BScN	St John’s Mercy Medical Center	St Louis, Missouri, United States of America	Research Coordinator		
Kim	Fowler		BScN	St John’s Mercy Medical Center	St Louis, Missouri, United States of America	Research Coordinator		
Katie	Krause		RN	St John’s Mercy Medical Center	St Louis, Missouri, United States of America	Research Coordinator		
Jackie	O`Brien		BScN	St John’s Mercy Medical Center	St Louis, Missouri, United States of America	Research Coordinator		
Marianne	Tow		BScN	St John’s Mercy Medical Center	St Louis, Missouri, United States of America	Research Coordinator		
John	Ma		PharmD	St John’s Mercy Medical Center	St Louis, Missouri, United States of America	Pharmacy		
Kaitlin MS	Moore		BS	St John’s Mercy Medical Center	St Louis, Missouri, United States of America	Pharmacy		
Yaseen	Arabi		MD	King Saud bin Abdulaziz University for Health Sciences and	Riyadh, Saudi Arabia	Site Investigator		
Abdulaziz	Al-Dawood		MD	King Saud bin Abdulaziz University for Health Sciences and	Riyadh, Saudi Arabia	Site Co-Investigator		
Haytham	Tlayjeh		MD	King Saud bin Abdulaziz University for Health Sciences and	Riyadh, Saudi Arabia	Site Co-Investigator		
Alaaeldien	Ghanem		MD	King Saud bin Abdulaziz University for Health Sciences and	Riyadh, Saudi Arabia	Site Co-Investigator		
Ahmad	Hassanien		MD	King Saud bin Abdulaziz University for Health Sciences and	Riyadh, Saudi Arabia	Site Co-Investigator		
Mohamed	Hegazy		MD	King Saud bin Abdulaziz University for Health Sciences and	Riyadh, Saudi Arabia	Site Co-Investigator		
Ashraf	El Sharkawi		MD	King Saud bin Abdulaziz University for Health Sciences and	Riyadh, Saudi Arabia	Site Co-Investigator		
Felwa	Bin Humaid		BSc	King Saud bin Abdulaziz University for Health Sciences and	Riyadh, Saudi Arabia	Research Coordinator		
Hala	Alanizi		RN	King Saud bin Abdulaziz University for Health Sciences and	Riyadh, Saudi Arabia	Research Coordinator		
Nadyah	Alanizy		RN	King Saud bin Abdulaziz University for Health Sciences and	Riyadh, Saudi Arabia	Research Coordinator		
Njoud	Al Bogami		RN	King Saud bin Abdulaziz University for Health Sciences and	Riyadh, Saudi Arabia	Research Coordinator		
Mohammed	Muhaidib		PharmD	King Saud bin Abdulaziz University for Health Sciences and	Riyadh, Saudi Arabia	Pharmacy		
Jawaher	Gramish		PharmD	King Saud bin Abdulaziz University for Health Sciences and	Riyadh, Saudi Arabia	Pharmacy		
Randa	Alsomali		BSc(Pharm)	King Saud bin Abdulaziz University for Health Sciences and	Riyadh, Saudi Arabia	Pharmacy		
Nora	Devera		BSc	King Saud bin Abdulaziz University for Health Sciences and	Riyadh, Saudi Arabia	Pharmacy		
Marjane	Villafranca		BSc	King Saud bin Abdulaziz University for Health Sciences and	Riyadh, Saudi Arabia	Pharmacy		
